# Charcot neuro-osteoarthropathy: a review of key concepts and an evidence-based surgical management algorithm

**DOI:** 10.3389/fcdhc.2024.1344359

**Published:** 2024-08-16

**Authors:** Miltiadis Argyropoulos, William Wynell-Mayow, Oscar Johnson, Radwane Faroug, Karanjeev Singh Johal, Rupinderbir Singh Deol, Atef Hakmi, Simon Mordecai

**Affiliations:** ^1^ East and North Hertfordshire NHS Trust, Stevenage, United Kingdom; ^2^ Imperial College Healthcare NHS Trust, Faculty of Medicine, Imperial College London, Stevenage, United Kingdom

**Keywords:** Charcot, surgery, management algorithm, diabetic foot, foot and ankle

## Abstract

Charcot neuro-osteoarthropathy (CNO), mainly as a result of diabetic neuropathy, is a complex problem which carries significant morbidity, and is an increasing burden on healthcare as demographics change globally. A multi-disciplinary team (MDT) is necessary to treat the multiple facets of this disease. The multifactorial and non-homogenous nature of this condition and its management, has prevented the development of comprehensive guidelines based on level 1 evidence. Although there is a trend to surgically treat these patients in tertiary centres, the increasing prevalence of CNO necessitates the capability of all units to manage this condition to an extent locally. This article conducted a thorough literature search of Pubmed and Embase from 2003 to 2023 including the following search terms; “Charcot” “neuroarthropathy” “diabetic foot” “management” “surgery” “treatment” “reconstruction”. The results of this review have been summarised and synthesised into an evidence-based algorithm to aid in the surgical decision-making process, and improve the understanding of surgical management by the whole MDT.

## 1 Introduction

In the UK, the most common cause for admission to hospital for patients who have diabetes is foot problems (1). Diabetes now affects 6% of the global population and meets the World Health Organisation (WHO) definition of an epidemic (2). The effects of diabetes on the foot cause significant and potentially devastating complications (3–9). Neuropathy is the hallmark pathology of the diabetic foot, which can lead to the destructive features of ulceration, infection and Charcot neuro-osteoarthropathy (CNO) (9). CNO is characterised by acute aseptic inflammation of the bones and joints in the foot and or ankle (10–12). Without early diagnosis and intervention Charcot foot can lead to deformity, ulceration and in severe cases amputation or systemic sepsis (4, 9, 13). These features represent a considerable clinical and economic burden to the healthcare system, with the cost in 2014-2015 estimated at between £837 million and £962 million in England (14). CNO not only significantly increases morbidity and premature mortality, but also has a large impact on activities of daily living (ADLs) (9, 15, 16). There is an additionally increasingly recognized mental health impact of CNO (15, 17).

### 1.1 Epidemiology

The reported incidence of CNO ranges between 0.3% and 0.85% annually amongst people with type 2 diabetes (18). CNO affects those with both type 1 and type 2 diabetes, only the age of presentation being distinct, 3rd/4th decade and 6th/7th decade respectively (19). People with CNO have an increased life-time risk of ulceration and amputation (13). Compared with CNO alone, those with CNO and established deformity, have a seven-fold increased risk of ulceration (13). A recent review has demonstrated 5-year mortality for CNO, foot ulceration, minor and major amputations to be 29.0%, 30.5%, 46.2% and 56.6%, respectively (20, 21). This combination of deformity, reduced mobility, frequent infection, and hospital admissions leads to significant impact on patients’ daily life, not only physically but psychologically, with the majority of patients requiring lifelong support (7).

### 1.2 Pathophysiology

The causes of CNO and its sequelae are multifactorial (5, 9, 22). There are currently two broadly accepted hypotheses regarding the pathogenesis of the disease, these being the neurovascular and neurotraumatic theories (5, 8, 23). The neurovascular theory describes a hyperaemic state caused by alterations in the sympathetic nervous system increasing venous pressure leading to a compromise of the soft tissue supporting structures in the foot and ankle leading to instability and collapse (10). This hyperaemic environment has also been hypothesised to directly affect bone resorption via increased delivery of osteoclasts and monocytes (11).

The basis of the neurotraumatic theory is repetitive microtrauma in a limb that has lost its protective sensation (10). The response to this trauma activates an acute inflammatory process with the upregulation of multiple pro-inflammatory cytokines, down regulation of anti-inflammatory cytokines and the upregulation of pathways involved in osteoclastogenesis (11, 12, 22, 24). The consequences of this acute inflammatory process coupled with a hyperglycaemic environment are far reaching, affecting not only bone health, but also soft tissue structure, via non-enzymatic glycosylation (10). Weight bearing in the presence of this dysfunctional sensory system perpetuates the microtrauma, increasing inflammatory cytokines and preventing the normal modulation of bone remodelling (22). Ultimately, excessive bone turnover with weakened soft tissue support, results in fracture, instability and architectural collapse (11, 12, 22, 24).

### 1.3 Clinical presentation & assessment

CNO follows a well described trajectory and can be classified by stage of the disease process by the Eichenholtz classification (25). Patients may present at any point in the natural progression of the disease process. Pain is rarely a presenting complaint due to neuropathy (9). Patients usually will notice swelling, erythema or deformity of the limb which will prompt them to seek medical attention (9). Diagnosis can be challenging to the non-specialist (26). A study of 230 patients with CNO showed that 48% of them were misdiagnosed initially, the most common misdiagnoses being cellulitis, deep vein thrombosis (DVT) and fracture or sprain (27). Chronic Regional Pain Syndrome (CRPS), gout, and tuberculosis have additionally been noted as potential CNO mimics (28). Delayed diagnosis and lack of appropriate management only increase the burden of disease and increase the risks of progression to deformity, ulceration, and potential limb loss (8).

Clinical evaluation will reveal swelling, erythema and colour in the early stages of CNO (25). On average there is a 3.3-degree centigrade temperature difference between affected and contralateral limb (29). Where bilateral CNO is suspected or CNO is suspected with contralateral limb amputation then ascending temperature gradients (toe-knee) may be helpful (30). The elevation test is a useful differentiator from cellulitis whereby the erythema of the foot will subside upon elevation in contrast to cellulitis (31). Serological markers of inflammation will help rule out differential diagnoses (26). The diagnosis must be reassessed regularly as both pathologies can coexist. Plain radiographs alone will often show changes, however, MRI may be required for the earlier stages of the disease (32). Where MRI is contraindicated such as in the presence of a pacemaker or other implanted metal devices SPECT-CT (Single Positron Emission Computed Tomography) or other nuclear imaging scan (scintigraphy) may help establish the diagnosis (30). Once the diagnosis and stage are established, it is important to determine which anatomical sites are affected, to guide management as described in the Brodsky classification (25, 33).

## 2 Methodology

The authors performed a thorough literature search using PubMed and Embase between 2003 and 2023. Search terms were: “Charcot” <OR> “neuro-osteoarthropathy” <OR> “neuroarthropathy” <OR> “diabetic foot” <AND> “management’ <OR> “surgery” <OR> “treatment” <OR> “reconstruction”. Abstracts were screened for relevance by the authors to limit articles to those relating to the key surgical treatment paradigms for Charcot. Where review articles contained articles not identified from our search, original source texts have been reviewed and included. Established principles and the latest evidence-based techniques were synthesized with the current surgical practice of the senior authors in order to generate section headings and the evidence-based algorithm.

## 3 Results

**Figure 1:**
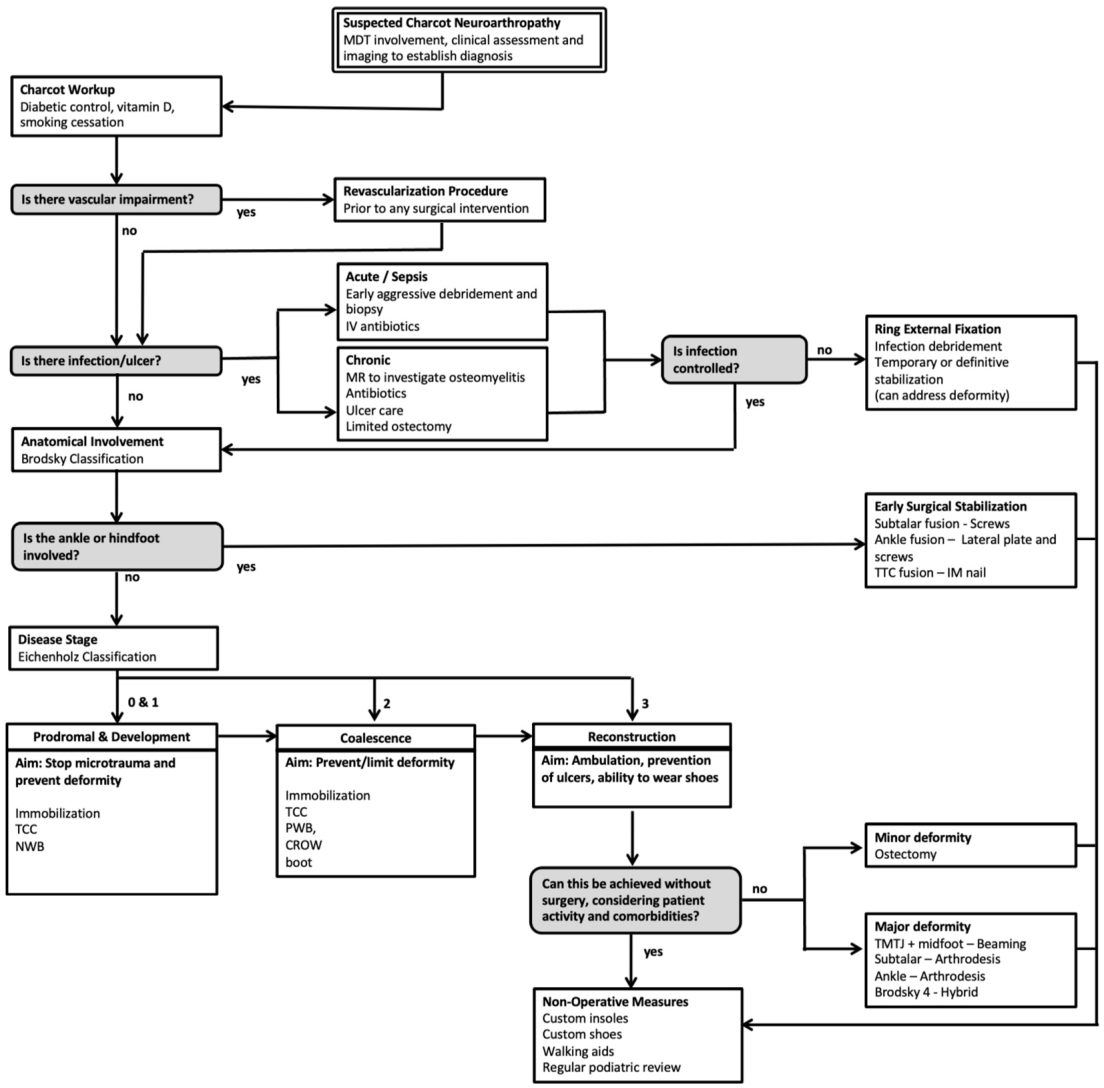
Evidence-based surgical algorithm proposed for the management of Charcot Neuroarthropathy (CNO).

This review article proposes a evidence-based evaluation and treatment algorithm for the surgical management of CNO (**Figure 1**). Sections 3.1 to 3.9 discuss the key surgical evidence which underpins the proposed management strategy. The overall surgical journey of a patient with suspected CNO according to this algorithm begins with an assessment of vascular impairment then exclusion of infection. Surgical management is then dictated by both the anatomical classification (Brodsky) and temporal staging (Eichenholz). The overall aim of the proposed algorithm (**Figure 1**) is to provide a shoeable foot free from infection.

### 3.1 Overall management and timing

There is a paucity of randomized controlled trials to inform the surgical management and timing of intervention in this complex problem (6, 8). A systematic review of 30 studies describing the management of a total of 860 patients was not able to offer conclusive recommendations on surgical interventions and their timing (34). More recently a systematic review including 42 studies (1116 feet) was unable to determine superiority between internal and external fixation techniques (35). The objectives of treatment will change as the patient progresses through the Eichenholz stages. If identified early in the fragmentation (or prodromal) stage, the aim of treatment is to protect the limb from microtrauma which initiated the pathological cascade by immobilizing the limb (33). Total contact casting (TCC) and non-weight bearing (NWB) have been shown to effectively reduce the fragmentation period (29, 36, 37). When the patient progresses to or presents in the coalescence stage, the aim is to prevent or limit deformity through further immobilization and off-loading depending on patient compliance (8). Once the resolution stage is reached the aim is to correct any resulting deformity in order to restore biomechanics for ambulation, prevent ulcer formation and to allow for the wearing of shoes (5, 6, 33). Surgical intervention has been described at all Eichenholz stages, however we advocate delaying surgery until the disease process has acquiesced in order to reduce the risk of postoperative wound complications with the exception of when there is ankle or subtalar involvement. At any stage infection may occur, however this is typically in stage 3 and is associated with an ulcer. Eradication will require long-term antibiotics, and surgical debridement may be necessary. Throughout the entire patient journey, the patient must be optimized with appropriate input from the MDT including consideration of psychosocial support (15, 17).

### 3.2 Patient optimization

The successful treatment of patients with CNO requires input from multiple specialists. National Institute for Health and Clinical Excellence (NICE) guidelines recommend the Multi-Disciplinary Team (MDT) must include an endocrinologist to control and manage diabetes, this is particularly important in the presence of infection (38). A target HbA1c < 7% should be sought and reconstructive surgery delayed in patients with HbA1c > 8% as higher levels are associated with a significantly greater risk of complications (39). Vitamin D levels and nutritional status are both relevant to bone metabolism and wound healing (9, 30). High rates of vitamin D deficiency have been demonstrated in patients with CNO posing a particular threat to bone quality required for surgical fixation (40). Podiatrists, plaster technicians and orthotists with training in TCC and ulcer management will provide input at every stage of the patient journey. NICE guideline 19 on diabetic foot problems advises against the use of bisphosphonates for CNO as does the International Working Group on the Diabetic Foot (IWGDF) (30, 38). Other previously proposed medical treatments including calcitonin, parathyroid hormone, methylprednisolone, and denosumab are additionally not recommended by the IWGDF on the basis of level 1 evidence from randomized control trials (41).

### 3.3 Revascularization

A vascular surgeon is a central member of the CNO MDT. CNO complicated by peripheral arterial disease has been shown to confer an increased risk of minor and major amputation and hospitalization, compared to CNO without vascular compromise (42). Limb perfusion should be assessed clinically upon presentation (8, 38, 43). Where there is either diagnostic uncertainty or clinical evidence of arterial insufficiency further assessment of foot perfusion should be undertaken (43). Ankle-brachial-pressure index has been shown to be unreliable in diabetic peripheral vascular disease due to arterial stiffness and calcification (44, 45). Continuous doppler waveform assessment may provide qualitative assessment of arterial perfusion with a monophasic waveform and loss of reverse flow highly suggestive of underlying arterial disease which may warrant revascularisation in the presence of persistent infection or if corrective surgery is being planned (45, 46). Duplex ultrasound, CT and MRI angiography all have utility in assessing the morphological distribution of peripheral artery disease prior to planning revascularisation (43, 44).

### 3.4 Infection

Once the diagnosis of CNO has been established, it is important to ascertain the presence of any infection or ulceration as this will affect management (9). CNO complicated by infection is associated with 12-fold higher risk of major lower extremity amputation (13). Infection will typically be secondary to ulceration, and this can arise at any stage of the disease process (47). In an acute infection where there is sepsis or diabetic foot attack this becomes the treatment priority (47). There is significant mortality associated with sepsis, even greater in people with diabetes, so it must be recognised early and treated as an emergency (48). Urgent debridement of infected or devitalised bone and soft tissues, along with deep tissue biopsy for microbiology cultures is necessary (48). Radical debridement should be protocolised using a Red-Amber-Green “RAG” categorisation of tissues to prevent under debridement (49). Broad spectrum empirical intravenous antibiotics as per local hospital protocols, and diabetic control are important in managing infection acutely (48). Monitoring of tissues and further debridement is often required in the diabetic foot attack (47, 49). Empirical antibiotics should be converted to targeted therapy on local microbiologist advice once culture results from intra-operative samples become available, and biochemical and clinical response should be monitored (38). Antibiotic eluting calcium preparations can be used to fill contained bone defects or by using the silo technique, in order to treat osteomyelitis (50, 51). Frequently, debridement will result in open wounds with significant soft-tissue loss (49). Specialist wound care such as negative pressure dressings or larva therapy may be prove helpful (52, 53). When infection is controlled, ulcers must be allowed to heal by granulation (54). In the situation where there is a large defect, or uncovered bone or tendon, early plastics input should be sought (55). Any subsequent casting must be appropriately padded or windowed to allow for wound care and off-loading of the affected area (56). If the infection is controlled but an ulcer has not completely healed, reconstruction with internal fixation can be considered provided there is no exposed bone (50).

### 3.5 Ankle or hindfoot involvement

Between 10 and 20% of cases involve the ankle and/or subtalar joints (4, 57). Disease in these joints is associated with high levels of instability with casting alone, resulting in early multiplanar deformity that can result in limb-length inequality (40). As such, early surgical intervention is recommended (58). If there is subtalar joint involvement a talocalcaneal arthrodesis can be undertaken, typically the valgus deformity will have to be corrected during joint preparation (59). If the ankle is involved, ankle arthrodesis is indicated using a plate and compression screw construct to maximise stability (59). Tibio-talo-calcaneal (TTC) arthrodesis may be required if both ankle and subtalar joints are involved, using a hindfoot nail (59, 60). A wider nail diameter has been shown to result in greater union rates and the use of a supplementary hindfoot compression screw has shown to improve union rate (95% versus 78%) (61). The overall complication rate in this subgroup of patients has been reported as 43% with approximately 30% incurring a superficial or deep infection (58). The use of allograft to recover lost height has been described, however this is associated with a higher complication rate, and we would therefore not recommend its use (62). In the presence of persistent infection or ulceration, a circular frame construct can be used (60).

### 3.6 Development

In this initial stage it is important to establish the diagnosis (9). The mainstay of treatment is to minimise any further microtrauma using a TCC to immobilise and off-load the limb (36). The duration of TCC will depend on response to treatment (36). This can be determined by clinical examination, temperature differential and MR if required (30). Typically, the fragmentation stage will last 2-4 months (9). Casts should be changed every 2-4 weeks to monitor for ulcers and to ensure the cast is well moulded (3). Arthrodesis in this stage has been described with positive results as it achieves immobilisation, however the view of the authors is that the risk of complications is too high (9).

### 3.7 Coalescence

Further protection is needed during coalescence in order allow fracture healing and limit displacement and deformity, with a particular focus on maintaining the foot arches (29). TCC and gradual increase of weight loading is advised under radiographic surveillance (8, 33). The mean duration of immobilisation of midfoot Charcot is 4-6 months until evidence of radiographic union and temperature equalisation (63). Charcot restraint orthosis walker (CROW) boot or regular boot have shown similar results if they are not removed (5, 30). The choice of protection will depend on patient compliance and disease progression.

### 3.8 Reconstruction

The management in this final stage can vary drastically depending on degree of deformity and anatomical site (60, 64). For minor deformity, custom made orthotics or shoes along with long-term podiatric support may be sufficient (65). In situations where there is minor deformity putting the foot at risk of ulceration or causing pain, ostectomy can be undertaken, either with open surgery or using minimally invasive techniques (57, 60, 64). Weight bearing CT can be helpful in determining the surgical targets (66). Achilles tendon lengthening can prove helpful in improving fixed flexion both in mild and severe deformity as well as calcaneal pitch and cuboid height (37). Recurrent ulceration can occur in up to 30% of patients treated with a TCC so it is imperative to identify surgical targets early to address areas at risk prior to the development of ulcers (67). Exostectomy alone was shown to result in ulcer healing in up to 60% of patients (7).

### 3.9 Deformity correction

**Figure 2:**
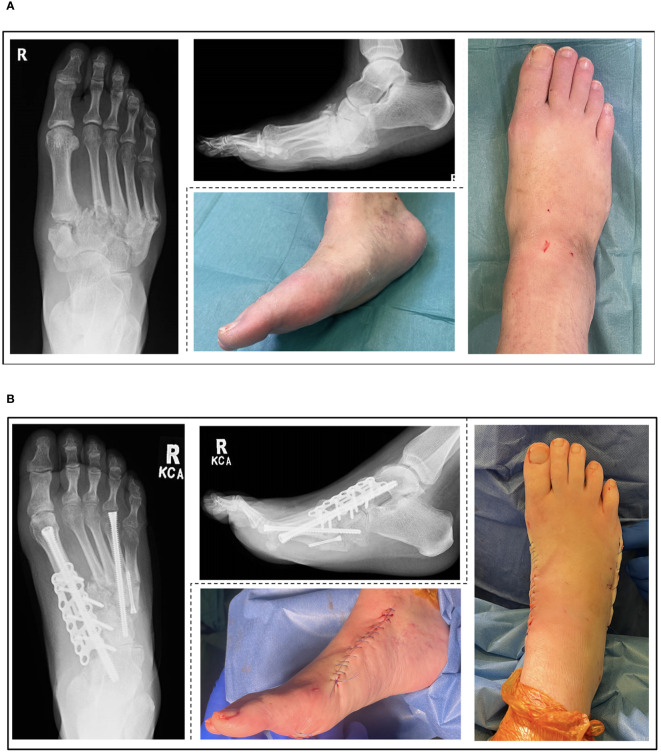
Pre-operative **(A)** and post-operative **(B)** radiographic and clinical images of an Eichenholz stage 3 (resolution), Brodsky type 1 (midfoot) Charcot Neuroarthropathy (CNO) treated according to the proposed algorithm.

Severe deformity which is not amenable to ostectomy alone will require a combination of ostectomy, osteotomy and arthrodesis (9,1 60, 64, 68). **Figures 2A, B** shows example radiographic and clinical images from a deformity correction performed at our unit according to the proposed treatment algorithm (**Figure 1**). This case demonstrates Eichenholz stage 3 and Brodsky type 1 CNO with significant deformity, not amenable to ostectomy alone. In the presence of infection or ulceration, a staged approach is required (50). External ring fixation may be used as a temporary stabilisation method while infection or ulceration are addressed, or as a means of definitive stabilisation and deformity correction (68, 69). External fixators can be effective however, they are poorly tolerated and fraught with complications, particularly in the group of patients with CNO (70). Whilst eradication of infection is imperative prior to internal fixation, ulcer healing is preferred rather than mandatory if it is distant from the surgical field (68). The techniques employed, depend on the anatomical site, and can be combined according to which joints are affected creating hybrid constructs (71). Delaying surgery in patients with severe deformity is associated with higher rate of soft tissue complications (76%) (72). Correction of deformity results in greater patient satisfaction when compared to ulcer management alone (73). The super construct concept aims to maximise stability across the fragmentation area (74). It differs from traditional orthopaedic principles in that the fusion zone extends beyond the affected joints utilising high rigidity device constructs to maximise stability, effectively bridging from healthy-to-healthy bone and bypassing the affected segment (74). Extruded bone must be removed or resected if not constructible in order to reduce risk of ulceration and decompress the soft tissue envelope (68).

The most common area to be affected is the midfoot, approximately 60% as per the Brodsky classification (25). The principles of surgery are to access the deformed joints using a dorsomedial and dorsolateral incision in order to reduce any subluxation and to debulk protruding bone and to fuse the affected joints (69, 75, 76). Commonly a wedge resection will be necessary to achieve this (69). Intramedullary fully threaded screws of at least 6.5mm diameter known as beams have been shown to have a lower failure rate compared to plates (77). Beaming achieves adequate compression for the purpose of arthrodesis along with restoration of medial and lateral column alignment and arch architecture (76). When performing screw fixations hydroxyapatite (HA) coated screws have been noted to reduce the risk of screw migration in poor quality bone (78). An osteotomy may be necessary if malunion has occurred (76). The joint surfaces are prepared for arthrodesis and a beam is passed retrogradely from the first metatarsal head intramedullary through the medial cuneiform, the navicular and into the head of the talus in order to restore the stability of the medial column (77). The construct can be augmented using a dorsomedial plate in a hybrid fixation (79). The lateral column stability is restored by similarly inserting a beam through the fourth metatarsal to the cuboid and if required the calcaneum (80). Complications have been shown to be higher when only a single column is stabilised and the Achilles tendon is not lengthened (81, 82). Postoperatively the patient typically remains NWB in a cast for 12 weeks, followed by gradual loading and reducing immobilisation (77, 83).In the situation where ulcer healing is not possible due to deformity, ring fixation has been described to have good results (69). Hybrid fixation methods have been described to yield good results whereby internal fixation is used in combination with a circular frame or monolateral external fixator (71, 75).

## 4 Discussion

This literature review has identified the key surgical concepts and evidence in the management of CNO. Overall this study found a paucity of randomised controlled trials or other level 1 evidence for the management of CNO (6, 8, 78). Much of the available evidence comes from retrospective studies, case series, and expert opinion. Based on a synthesis of the available evidence and the authors’ experience we propose a pragmatic management algorithm for CNO (**Figure 1**). Management is based on the stage of presentation (Eichenholz), the presence or absence of infection, the presence or absence of vascular insufficiency, and the anatomical location of the disease (Brodsky). This algorithm provides clinicians with an evidence-based guide to surgical treatment in this complex condition, aimed at minimising complications, and improving outcomes. Patients with suspected CNO should undergo a detailed medical and surgical evaluation to clarify diagnosis early and identify complicating issues such as vascular impairment and infection. Early stage CNO should be managed with offloading through reducing weightbearing and TCC. Late stage CNO should be treated according to the deformity present in order to provide a shoeable foot, free of ulcers and at-risk areas in order to restore the patient’s ADLs maximising function and quality of life.

## 5 Conclusion

CNO is a complex disorder associated with significant morbidity. There are multiple factors to consider in the management of CNO such as patient optimisation, vascularity, infection, anatomical involvement, stage of disease, severity of deformity, timing of surgery and patient preference or compliance. The presented evidence-based surgical treatment algorithm can be used as a roadmap to aid decision making and management, in particular, by clinicians not working in a tertiary centre where dedicated diabetic foot MDTs may not be established.
